# Supplementary material for: Forbidden, yet common: Female genital cutting among the Oromo in central Ethiopia
Source: PLoS One. 2024 Dec 17;19(12):e0315566. doi: 10.1371/journal.pone.0315566 (PMC11651596; doi:10.1371/journal.pone.0315566)
Supplement: S1 File — (DOCX) [file pone.0315566.s001.docx]

**Cases**

**Case one**

**Interviewee: 54**

A father who has attended higher education has no interest in cutting his daughter’s genitalia because he knows the negative implications of it. His wife, of course, did not go to formal school. His daughter, who was by then eleven years of age, asked her father to organize her surgery to remove her clitoris. If not, she would not go to school anymore. She justified that since she did not get her genitals cut, she was ashamed of being with her peers whose genitals had already been cut. The father tried to convince her that she was lucky because her body had not been cut unnecessarily, whereas it is unfortunate that her peers’ genitals had been cut for that would have some negative medical implications. He tried to convince her repeatedly, yet his daughter was not willing to give up her request. Even though the father knows the negative impact of FGC and insisted not to perform it, the girl strongly demanded to undergo her genital cutting; otherwise, she would decide not to go to school anymore. Her demand to get cut her genital was now and then. Finally, he allowed his daughter to undergo the demanded genital cutting. The girl is his most loved child and has close relations with him compared to her four brothers. He suspected that the demand might have come from the mother via the daughter. Yet, the mother did not support the girl overtly. She did not also arrange genital cutting for her daughter. She might be, as the father said, the sponsor of the request of the girl who put pressure on her father to accept her demand.

**Case two**

**FGC 36 (one of the discussants narrated it)**

She heard the story from an old woman about 60 years of age. Two young men went to the nearby town with their respective girlfriends. The young men were friends and shared every secret. The girlfriend of the first man had undergone her genital cut, and the second girl had not. Both men went to bed with their girls overnight. In the morning, the man with the girl whose genitalia had not been cut asked his friend how his night was. The second man responded that he was fine with his girlfriend, and they enjoyed the night. However, his friend complained that he had a problem with his girl. Since her clitoris had not been cut, he felt discomfort during sexual intercourse. The girl also recognized that her boyfriend was not happy but did not know the reason behind it. Back home, she consulted one of her female relatives. The woman told the girl that the problem was with her genitalia, which had not been cut. And the blame was directed at the mother of the girl for letting her daughter remain with her children.

**Case three**

**Interviewee 40**

Our key informant knows that there was a woman married and divorced three times. She married and stayed short with her husband. He complained that they did not fit sexually. She married for the second time. Still, she divorced soon. Once again, she married for the third time, but their marriage was concluded in divorce. All of them complained that she had not undergone genital cutting. Our key informant strongly argued in favor of FGC. Because she believed that the complaint of all three men referred to the high sexual demand of the woman as well as inconvenience during sexual intercourse.

**Case Four**

**Interviewee 38**

I lost my parents when I was a high school student. I am an elder child of the family and took on the responsibility of bringing up my younger brothers and sisters. I did not let my younger sisters cut their genitals. I had no complaint about their sexual behavior toward my sisters. For instance, one of my sisters is so modest and a married virgin. Now, she is a mother of two and leading such a successful marital life. Her husband loves her too much.

**Interviewee 44**

**Case Five**

There was a woman brought up by her grandmother who was not able to arrange genital cutting for her granddaughter. As she became mature, the girl got married and started leading a lovely marital life. However, at a certain point, her husband just abandoned her and went somewhere unknown. The woman who was on good terms with her husband was abandoned suddenly without any problem she knew. She complained that she missed her husband in divorce because her genitalia was not cut. Of course, she did not hear any complaints related to sexual matters from her husband. In the meantime, she did not know what made him abandon her. She simply guessed that the problem might be with her uncut genitalia. She knew that her husband had already had the information that she had not undergone genital cutting. The woman narrated the story to our informant in person, and she was in search of a solution, including if the surgery was possible at that stage. However, our informant did not know what happened next.

**Case Six**

**Interviewee: 44**

There is a woman whose mother is economically weak and was not able to organize a genital cutting for her daughter. The mother gave birth to this girl as a widow after the death of her husband. Children from her late husband called their younger sister from a different father *diqalaa* (out of wedlock). As she grew up and knew that her genitalia was not cut, she extremely blamed her mother. She also frequently requested her mother arrange a session for cutting her genital. However, she was not successful. She took the act as negligence as well as segregation, for she was from a different father. It is a continuation of the harassment she suffered from her half-sisters and brothers. Leaving her family behind, she left for another place in despair, where she married and gave birth to a child. Nobody knew her relations with her husband. Yet she was not happy and lived short.

**Case Seven**

**Interviewee: 46**

A father who attended grade ten and is now a farmer has two young daughters. He is also conscious of the negative impact of FGC. His wife, who has not attended a formal school, believes that FGC is mandatory and necessary for the cleanliness of women, sexual modesty, and cultural conformity. She believes that a woman who has not undergone genital cutting has something unwanted ‘dirty’ on her genital. She never wants to negotiate, even with her husband, whether she would perform genital cutting of her daughters or not. She believes that taking care of girls is the responsibility of mothers. Mothers must bring up their daughters as per the custom of their society. If the mothers let their daughters remain with their clitoris, it is shame on them; fathers are not directly responsible for this. Thus, she organized a secret genital cutting session for her young daughters without the knowledge of her husband. She assigned her sister-in-law as a *jaala (sponsor*) to maintain the confidentiality of the case. Since the chosen *Jaala* has also the same attitude towards FGC, she cooperated with her sister-in-law. The father recognized lately that his daughters had undergone FGC. He saw the girls were under treatment for their wounds.

**Case Eight**

**Interviewee: 54**

The father is an administrator of a *kebele* (local rural administration). He also attended formal school. He attended several rounds of training and awareness-creation workshops as an administrator. He is also in charge of creating awareness at the local level, in his *kebele*. He has two daughters and two sons uncircumcised. At one point, he organized a circumcision ceremony for his sons. The sons were circumcised at the health center for safety. On the same day, he traveled to attend a two-day meeting at one of the nearby district towns. The circumcision of the boys was handled by their respective *jaala* (sponsors)*.* Accordingly, when the two sponsors of the boys returned home from the clinic, there were two women at home with the same status. They performed the FGC of two young girls at home and joined the two men coming from the nearby clinic after the successful circumcision of boys. The absence of the father was an attempt to convince others that the act of FGC in his family was outside of his knowledge. However, it was intentional, and pretending to escape the possible legal accountability both as an administrator who oversees controlling FGC and as a father whose daughters were exposed to injuries for no medical reason.

**Case Nine**

**Interviewee: 44**

A woman was working in the Office of Women, Youth, and Children of Dawo District. She is one of the experts to creates awareness of the negative implications of FGC and implements any breach of the rule prohibiting FGC. However, an informal report came to the head of the office and the office of police that she had cut the genitals of her two daughters. The two offices attempted to check if the report was true. The small girls did not confirm the act. The girls were taken to the clinic to check whether or not they had been cut. There was also no medical evidence indicating the act was done. The parents insisted that the girls have their genitals untouched. The former head of the office suspected that the medical evidence was not reliable or might have been reported in favor of the parents to protect them from being accused of the act. The point was not whether they would be punished by some amount of money, but the meaning it gave to the wider public. The woman, who by herself was teaching about the negative implications of the act, was not expected to commit the same wrong.

**Case Ten**

**Interviewee 51**

The couples agreed and performed the genital cutting of their daughter secretly. The cutting was done by less skilled local practitioners. The injury was infected and serious. They could not take the victim to the health center because the act of cutting was illegal and the parents and other participants might be exposed to legal prosecution. They wanted to keep the confidentiality of the practice. They kept the child at home for long without any medical treatment. Finally, they took her to a private clinic. Fortunately, she survived the infection.

**Case Eleven**

**Interviewee 50**

In Busa, which is the administrative center of the district, the police controlled a practitioner, parents, and sponsors. As usual, the victims were taken to the clinic for medical treatment. Since there was nowhere to stay for the children, the district court took a quick decision the next morning in which the practitioner was punished with 2000 birr, and the mother and the two sponsors (jaala) were punished with 500 birr each.

**Case Twelve**

**Interviewee: 44**

Usually, circumcision is organized in the week after the Ethiopian Easter day. This is so because most of the followers of Ethiopian Orthodox Christianity do not slaughter for two months before Easter. After the ceremony, there is a feast to entertain the guests. Taking into consideration the Office of Women, Youth, and Children scanned the potential FGC in the district. One of the known women as FGC practitioners who reside in Gaba Jimata *kebele* was kept under the strict supervision of the police. However, another woman from another locality took the role and performed FGC over the areas moving from place to place. The office got information that FGC was going on in mass in the district. Finally, the office, in cooperation with the police, got two girls whose genitals were cut in one home. As usual, the office took the girls to the nearby clinic for medical treatment and arrested the parents and the sponsors. Each of them paid a fine of 500 Ethiopian Birr.

**Case Thirteen**

**Interviewee 44**

The Office of Women, Youth, and Children got information from local people that FGC was performed at Qarsa Kebeles in the Dawo district. The office, in cooperation with the office of the police, arrived at the place soon and controlled the actors red-handed. Two girls five and seven years of age were injured. The girls whose genitals were cut were taken to the clinic for medical treatment and to make sure that they were safe. Then, the case was taken to the court, and all the actors—the parents, the sponsors, and the practitioner—were punished with money. The parents and the two sponsors were punished with 500 Birr each, and the practitioner was punished with 2000 Birr. The latter was aged, a woman of more than 60 years of age. The court took into consideration her age and decided to punish her with a fine. The decision was in line with the Ethiopian criminal code Article 565, which states that “whoever circumcises a woman of any age is punishable with simple imprisonment for not less than three months or a fine not less than five hundred Birr.
