# Supplementary material for: Forbidden, yet common: Female genital cutting among the Oromo in central Ethiopia
Source: PLoS One. 2024 Dec 17;19(12):e0315566. doi: 10.1371/journal.pone.0315566 (PMC11651596; doi:10.1371/journal.pone.0315566)
Supplement: Attachment — Submitted filename: PONE-D-24-24076.AK_pdf.pdf [file pone.0315566.s002.pdf]

PLOS ONE

Forbidden, yet common: female genital cutting among the Oromo in central Ethiopia

--Manuscript Draft--

**Manuscript Number:**	PONE-D-24-24076
**Article Type:**	Research Article
**Full Title:**	Forbidden, yet common: female genital cutting among the Oromo in central Ethiopia
**Short Title:**	Female Genital Cutting
**Corresponding Author:**	Dejene Gemechu Chala, Ph.D<br>Jimma University<br>Jimma, Oromia ETHIOPIA
**Keywords:**	female genital cutting; sexual behavior; legislative approach; Oromo; patriarchy
**Abstract:**	This article answers the question 'why female genital cutting (FGC) has remained common among the Oromo in central Ethiopia when it is legally forbidden.' Data for this article were generated through key informant interviews and focus group discussions. Our findings indicate that the government tries to use legislative and health approaches in its efforts to terminate FGC. However, FGC has survived over time because of the strong resistance that the community in the research area wages against the intervention efforts. The very reason behind the resistance to end FGC is a patriarchal value that shapes gender relations, women's sexuality and behaviors. The community gives precedence to the socio-cultural values of FGC over its criminalization and the stated negative health effects. Paradoxically, women are at the forefront in promoting FGC. They believe that FGC shapes the sexual behavior of girls and defines the gender identity of their daughters, while they believe that it is women's responsibility to raise girls and mold them into ideal female behavior.
**Order of Authors:**	Zerihun Mekuria Tesfaye, MA
	Dejene Gemechu Chala, Ph.D
	Jira Mekonnen Choroke, PhD
**Additional Information:**	
**Question**	**Response**
**Financial Disclosure**<br><br>Enter a financial disclosure statement that describes the sources of funding for the work included in this submission. Review the submission guidelines for detailed requirements. View published research articles from *PLOS ONE* for specific examples.<br><br>This statement is required for submission and **will appear in the published article** if the submission is accepted. Please make sure it is accurate.	The author(s) received no specific funding for this work.

Powered by Editorial Manager® and ProduXion Manager® from Aries Systems Corporation

**Funded studies**<br>Enter a statement with the following details:<br>• Initials of the authors who received each award<br>• Grant numbers awarded to each author<br>• The full name of each funder<br>• URL of each funder website<br>• Did the sponsors or funders play any role in the study design, data collection and analysis, decision to publish, or preparation of the manuscript?<br><br>Did you receive funding for this work?	
**Competing Interests**<br><br>Use the instructions below to enter a competing interest statement for this submission. On behalf of all authors, disclose any competing interests that could be perceived to bias this work—acknowledging all financial support and any other relevant financial or non-financial competing interests.<br><br>This statement is required for submission and **will appear in the published article** if the submission is accepted. Please make sure it is accurate and that any funding sources listed in your Funding Information later in the submission form are also declared in your Financial Disclosure statement.<br><br>View published research articles from *PLOS ONE* for specific examples.	there is no competing interest

**NO authors have competing interests**<br><br>Enter: *The authors have declared that no competing interests exist.*<br><br>**Authors with competing interests**<br><br>Enter competing interest details beginning with this statement:<br><br>*I have read the journal's policy and the authors of this manuscript have the following competing interests: [insert competing interests here]*<br><br>* typeset	
**Ethics Statement**<br><br>Enter an ethics statement for this submission. This statement is required if the study involved:<br><br>• Human participants<br>• Human specimens or tissue<br>• Vertebrate animals or cephalopods<br>• Vertebrate embryos or tissues<br>• Field research<br><br>Write "N/A" if the submission does not require an ethics statement.<br><br>General guidance is provided below. Consult the submission guidelines for detailed instructions. **Make sure that all information entered here is included in the Methods section of the manuscript.**	Jimma university, College of Social sciences ad Humanities

**Format for specific study types**<br><br>**Human Subject Research (involving human participants and/or tissue)**<br>• Give the name of the institutional review board or ethics committee that approved the study<br>• Include the approval number and/or a statement indicating approval of this research<br>• Indicate the form of consent obtained (written/oral) or the reason that consent was not obtained (e.g. the data were analyzed anonymously)<br><br>**Animal Research (involving vertebrate animals, embryos or tissues)**<br>• Provide the name of the Institutional Animal Care and Use Committee (IACUC) or other relevant ethics board that reviewed the study protocol, and indicate whether they approved this research or granted a formal waiver of ethical approval<br>• Include an approval number if one was obtained<br>• If the study involved *non-human primates*, add *additional details* about animal welfare and steps taken to ameliorate suffering<br>• If anesthesia, euthanasia, or any kind of animal sacrifice is part of the study, include briefly which substances and/or methods were applied<br><br>**Field Research**<br><br>Include the following details if this study involves the collection of plant, animal, or other materials from a natural setting:<br>• Field permit number<br>• Name of the institution or relevant body that granted permission	
**Data Availability**<br><br>Authors are required to make all data underlying the findings described fully available, without restriction, and from the time of publication. PLOS allows rare exceptions to address legal and ethical concerns. See the PLOS Data Policy and FAQ for detailed information.	No - some restrictions will apply

Powered by Editorial Manager® and ProduXion Manager® from Aries Systems Corporation

A Data Availability Statement describing where the data can be found is required at submission. Your answers to this question constitute the Data Availability Statement and **will be published in the article**, if accepted.<br><br>**Important:** Stating 'data available on request from the author' is not sufficient. If your data are only available upon request, select 'No' for the first question and explain your exceptional situation in the text box.<br><br>Do the authors confirm that all data underlying the findings described in their manuscript are fully available without restriction?	
**Describe where the data may be found in full sentences. If you are copying our sample text, replace any instances of XXX with the appropriate details.**<br><br>• If the data are **held or will be held in a public repository**, include URLs, accession numbers or DOIs. If this information will only be available after acceptance, indicate this by ticking the box below. For example: *All XXX files are available from the XXX database (accession number(s) XXX, XXX.*).<br>• If the data are all contained **within the manuscript and/or Supporting Information files**, enter the following: *All relevant data are within the manuscript and its Supporting Information files.*<br>• If neither of these applies but you are able to provide **details of access elsewhere**, with or without limitations, please do so. For example:<br><br>*Data cannot be shared publicly because of [XXX]. Data are available from the XXX Institutional Data Access / Ethics Committee (contact via XXX) for researchers who meet the criteria for access to confidential data.*<br><br>*The data underlying the results presented in the study are available from (include the name of the third party*	The data are available with the researchers and may be provided upon requests

Powered by Editorial Manager® and ProduXion Manager® from Aries Systems Corporation

and contact information or URL).<br>• This text is appropriate if the data are owned by a third party and authors do not have permission to share the data.<br><br>* typeset	
Additional data availability information:	

Powered by Editorial Manager® and ProduXion Manager® from Aries Systems Corporation

Manuscript
Click here to access/download;Manuscript;FGC draft manuscript, June 7 2024.docx

Forbidden, yet common: female genital cutting among the Oromo in central Ethiopia

Zerihun MekuriaTesfaye [1¶], Dejene Gemechu Chala[1*], Jira Mekonnen Choroke[1&]

[1]Department of Social Anthropology, College of Social Sciences and Humanities

* corresponding author: E-mail: achekebo@gmail.com

[¶]First authors

[&] Second Author

Abstract

This article answers the question 'why female genital cutting (FGC) has remained common among the Oromo in central Ethiopia when it is legally forbidden.' Data for this article were generated through key informant interviews and focus group discussions. Our findings indicate that the government tries to use legislative and health approaches in its efforts to terminate FGC. However, FGC has survived over time because of the strong resistance that the community in the research area wages against the intervention efforts. The very reason behind the resistance to end FGC is a patriarchal value that shapes gender relations, women's sexuality and behaviors. The community gives precedence to the socio-cultural values of FGC over its criminalization and the stated negative health effects. Paradoxically, women are at the forefront in promoting FGC. They believe that FGC shapes the sexual behavior of girls and defines the gender identity of their daughters, while they believe that it is women's responsibility to raise girls and mold them into ideal female behavior.

Key terms*: female genital cutting, sexual behavior, legislative approach, Oromo, patriarchy*

Introduction

This article focuses on female genital cutting (FGC) among the Oromo in central Ethiopia. FGC includes all the surgical procedures for partial or total removal of external female genitalia and other injuries to the female genital organs for no medical reasons. It ranges from a minor incision at the tip of the clitoris to a total excision of the external genitalia and stitching of the vaginal opening [1, 2]. This article, thus, addresses the question of how and why this practice,— FGC, — is legally forbidden and stated to be harmful in terms of health, and a practice that violates human rights has remained common in the study area. The community challenges the criminalization of FGC and other interventions by the government, mainly by maintaining the confidentiality of the practice and undermining the penalties that result from the practice.

The study was conducted in Dawo District, which is situated in central Ethiopia. The administrative center of the district is located approximately 90 kilometers southwest of Addis Abeba. According to the CSA population projection [3], the total population of the district is 122,731 people. CSA [4] shows that the Oromo constitute 93.35 percent of the population, while Afaan Oromo is both the official and spoken language of the district. Ethiopian Orthodox Christianity is practiced by more than 90% of the population. There are followers of traditional beliefs as well as of Protestant Christianity. A recent publication by Gemechu and Mekuria [5], which focuses on the Dawo district, shows that more than 90% of the schoolgirls who responded to their questionnaire have already undergone FGC. UNICEF [6] also shows that the prevalence of FGC in the Oromia National Regional State, where our research site is situated, is between 76% and 90% for women between the ages of 15 and 49.

The World Health Organization (WHO) estimates that globally, more than 200 million girls and women are affected by FGC, while an additional three million girls are at risk of having

their genitalia cut every year. The magnitude greatly varies across regions. The high concentration of this practice is in some African, Asian, and Middle Eastern countries [7, 8]

In Ethiopia, as in other African countries and beyond, FGC is common. Llamas [9] shows that 24 percent of Ethiopian girls between 0 and 14 years of age have undergone some form of FGC. About 74% of the women between the ages of 15 and 49 have already had their genitals cut. The Ethiopian Demographic Health Survey (EDHS) [10] shows that 65 percent of Ethiopian women aged 15–49 have already undergone the surgery, while the percentage ranges between 91 and 100 for Somali and Afar regions [6, 11].

Nonetheless, FGC is a criminal act in Ethiopia. The Ethiopian Criminal Code [12] states that "whoever circumcises a woman of any age is punishable with simple imprisonment for not less than three months or a fine of not less than five hundred Birr." Article 566 (1) reads: "Whoever infibulates the genitalia of a woman is punishable with rigorous imprisonment from three to five years." Ethiopia introduced a National Strategy and Action Plan to eliminate ‘harmful traditional practices’ (HTPs, Early marriage, FGC, child preference, wife inheritance, abduction, uvulectomy, milk tooth extraction, are all examples of HTPs) against women and children in 2013. One of these practices is female genital cutting (FGC) (Federal Democratic Republic of Ethiopia, Ministry of Women, Children, and Youth [FDRE, MoWCY] [13], European Union Agency for Asylum (EUAA), [14]. Despite the national legislation being in place, the practice has continued mainly because of some challenges around knowledge and law enforcement. Local community leaders strongly support the continuity of the practice, and many local officials value the social norm of FGC more than they value the law banning it [14].

However, the question of whether or not the legal enactment of the prohibition of FGC as well as the strong campaign against it are effective in ending the practice demands due consideration. Thus, this article aims to explain why FGC remains a common practice among the Oromo in

Dawo District, Oromia National Regional State, Ethiopia. To answer this question, the article tends to show that the community gives precedence to the cultural values of the FGC over its health effects and criminalization. It also addresses ways to withstand the potential legal prosecution that results from the FGC. We argue that patriarchal system is behind the scene to promote FGC in this research area. Below, we briefly discuss some concepts related to this practice to situate FGC in the area in the wider literature and to show the types of FGC that are prevalent in this area. We argue that patriarchal system is behind the scene to promote FGC in this research area.

FGC: A conceptual definition

FGC as a cultural practice takes many forms, even within the same community and society. There are numerous terminologies for FGC. These are "female genital mutilation," "female genital cutting’, "female circumcision," "female genital surgery," “female genital modification,” and so on [1, 15]. The multiplicity of terminology indicates the complexity of the problems related to FGC. These different terminologies reflect the different positions or contexts in which the issue is being understood [1]. Debates around the terms and the meanings they convey are ongoing [15]. However, according to Llamas [9], the use of the term "female circumcision" received grave criticism for tending to normalize the act by comparing it with male circumcision, which is a widely accepted practice. Since the 1970s, feminist activists have exposed the harmful consequences of this tradition and began to use the term "mutilation" (FGM) instead of "circumcision." On the other hand, some countries insist on using the term "female circumcision," since the term "mutilation" connotes offensive and evil intent. "Female genital cutting" is also another alternative term used in the literature.

At our research site, *dhaqnaqabaa*, 'circumcision' is a word that is used to refer to female genital cutting. The official Ethiopian legal tools related to the issue also use the same term. Our target population practices what is commonly known as clitoridectomy, that is, the removal of part of the clitoris. In Ethiopia, there are four forms of FGC: clitoridectomy, excision, infibulation, and all other harmful procedures. Infibulation is more severe and common among the Somalis and Afar. In Oromia, clitoridectomy is common [6, 16]. In this article, we use the term "female genital cutting" (FGC) to be less judgmental about this culturally-sensitive term [17]. The following section focuses on some of the common values behind FGC and the approaches/efforts that are being used to terminate it.

The Cultural Values of FGC

There are a variety of explanations behind the practice of female genital cutting. Some of these explanations have socio-cultural, hygienic, aesthetic, religious, and psycho-sexual reasons. The practice also serves as a rite of passage that turns girls into women by removing part of their genitals, the clitoris, which has the appearance of a male sexual organ, the penis. It serves as one way of ensuring a girl's gender identity [18, 19, 20].

Sally [15] and Diop et al [21] explain that patriarchy provides the cultural and institutional framework for the perpetuation of FGC. It is a reflection of the power inequality between men and women, in which men control women's sexuality, their reproductive systems, and their health. In short, FGC persists because of patriarchy. Pertinent to this, according to Morrison [22], the concept of "clitoral economy" shows the symmetry between the female body (the clitoris) and the male body (the penis). The author shows how the clitoris is vested with so much power in symbolic and semiotic constructions of the female body. Clitoris has been viewed as a threat to the hegemonic phallic economy in patriarchal societies where FGC is common.

In this regard, Sally [15] shows the paradox behind FGC in patriarchal societies. Women who are victims of FGC defend and promote it as part of their tradition, which marks a rite of passage as well as a cosmetic procedure with some aesthetic values. According to Ahan [23], in some African cultures, purity, which is related to FGC, is taken as a precondition to marriage. The second reason is to attain feminine behavior. The author gave the example of Somalia where FGC is mandatory and any girl who has not been exposed to it will never marry.

In traditional African culture, older women reproduce patriarchal traditions by imposing themselves on younger girls, the same way they were oppressed once. In this regard, women adjust themselves to be active agents within the patriarchal system to promote the system that has suppressed them [24, 25]. However, some studies show how the role of women in sustaining FGC changes across socio-demographic factors. Studies by El-El-Dirani et al. [26, 27, 28] show the associations between FGC and parental factors, particularly maternal education, age, FGC experience, and residence setting. The more mothers are educated, young, and have undergone FGC, the less likely it is that their daughters will also experience FGC.

Approaches to ending FGC

The UN, WHO, UNICEF, and other women's rights advocacy organizations have used four main approaches to end FGC. These are bodily and sexual integrity; human rights; legislative; and health approaches [29]. Feminist writers who advocate a bodily and sexual integrity approach emphasize women's sexual rights and satisfaction. This approach underlines how FGC impairs women's right to sexual health and pleasure by shaping, mediating, and controlling women's sexual enjoyment through social institutions [30]. In this regard, functionally, the clitoris is a site for sexual pleasure and is taken as a symbol of female sexuality by Western "second-wave feminists," and the removal of this part of women's genitalia

obliterates their sexual enjoyment, freedom, and expression [29]. In this regard, FGC is also linked to the need to reduce promiscuity and maintain premarital virginity [29, 31, 16].

However, some challenge the bodily and sexual integrity approach because they believe that sexual enjoyment must be "normal" and ethical. According to Brown et al. [29], since sexuality is guided by societal norms and is a private issue, conservatives challenge the Western prescription to stop FGC. In conformity with this view, Mutashi and Sass [32] reported that about 30% of Kenyan women are in favor of the continuation of FGC to preserve virginity.

The second is the human rights approach, which considers FGC as a basic violation of the human rights of girls and women. According to this approach, FGC harms females' bodies for no medical reason, and it is gendered discrimination and a fundamental violation of the human rights of women and children, including the right to freedom from discrimination, torture, and violence; the right to health; the right to education; and the right to life. This approach is prominent with the UN, the European Union, and several governments of developed countries [29]. FGC violates the rights of innocent girls who are not able to consent to the practice and has to end it [8, 16]. This position is what is usually known as the "zero tolerance" approach of the agencies [1].

However, there are also challenges to the human rights approach for it is biased toward the liberal democratic tradition that gives priority to individual rights instead of "community" rights [32]. For instance, Africans perceived the 1960s and 1970s European and American feminists' campaign to eliminate FGC as an imposition from the outside and another attempt at colonial imperialism [29].

The legislative approach is the third-to-end FGC. Since the turn of the century, governmental, intergovernmental, and non-governmental organizations, as well as academia, have made FGC a hot topic. Most of the countries where FGC is common have laws that prohibit the practice [15, 33]. For instance, the majority of African countries have laws that criminalize

FGC. The African Charter on the Rights and Welfare of the Child is another effort to abolish the practice [15]. These laws have labeled FGC as a criminal act of bodily harm or injury and provided a structural framework or "enabling environment" to act against it [29].

However, the debate is whether it is possible to end FGC by criminalizing the practice or not. There is a consensus that the law alone does not serve the purpose. Despite the criminalization of the act, local practitioners continue to cut female genitals secretly. This practice has contributed to even more unsanitary and unsafe conditions during the surgery [19, 15]. According to Brown et al. [29], there is another negative side to the legislative approach for parents who practice FGC underground, and it is less likely that they seek health care for complications resulting from the surgery for fear of prosecution.

The health approach is the fourth one, which emphasizes the negative health implications of FGC [15]. The WHO focused on health hazards to strongly justify the abolition of FGC. However, later, UN agencies shifted their attention from bodily injuries and therapeutic risks of FGC to the human rights violations in their efforts to end it [1, p. 7] Written sources show that the practitioners include midwives, trained circumcisers, doctors and nurses, or traditional practitioners—local specialists (circumcisers), traditional birth attendants, traditional healers, and experienced grandmothers—without any formal training. Regardless of who the practitioner is, she or he does the surgery mostly without anesthesia, antibiotics, or sterile equipment [9, 32]. Waigwa, et al. [8] divide the health effects of the practice into short- and long-term impacts. The first include severe pain, shock, bleeding, tetanus or bacterial infection, urine retention, open sores in the genital region, and injury to nearby genital tissue. The long-term consequences include recurrent bladder and urinary tract infections, cysts, infertility, an increased risk of childbirth complications, and the need for later and repeated surgeries for childbirth and sexual intercourse.

However, this is not without controversy. Some challenge this approach, for it does not take into consideration the lived realities of women who have experienced different forms of FGC [29]. In some cases, the community has considered FGC an important surgery. For instance, the Nigerians and the Ghanaians believe that FGC cures infertility, and for some Sudanese, it prevents diseases in infancy and promotes child health [15].

Despite the fact that the above approaches are all relevant to put an end to FGC, the local government at our research site uses the legislative and health approaches more vividly. The Ethiopian criminal code [12] labels FGC as a criminal act and a violation of the human rights of women and girls. There are also various government offices working against FGC and other cultural practices that are categorized as harmful. These are the Women, Youth, and Children Affairs (WYCA), Culture and Tourism, Health Office, Education, and Police offices. Despite this, FGC survived as a longstanding cultural practice. In the following sections, right after methods and materials, we will look at the cultural meanings of FGC as well as the factors that contribute to its persistence in the face of the government's efforts to end it in the research site.

Methods and Materials

This article is based on primary and secondary data. We used secondary data, such as legal codes, policies, and strategies to show some government efforts to stop FGC. However, the article is based primarily on qualitative data that were drawn from the field through key informant interviews and focus group discussions, which were conducted between February 27 and March 20, 2020. All the participants were purposively selected in consultation with the office of the WCYA of the district. Partly convenience sampling was used to identify the participants. The selection criteria were individual knowledge and access to information about the issue under discussion.

Key informant interviews

We interviewed a total of 20 key informants—13 women and seven men. The participants were three (a man and two women) from the Women, Youth, and Children Affairs Office; a focal person from the Police Office (a woman); an expert from the Culture and Tourism Office (a man); a woman expert from the Education Office; four women and three men from the community; and seven schoolteachers (five women and two men); three of the five women are married and have children and the remaining were bachelors, while both men are fathers who have daughters. The offices listed were chosen because they have directly or indirectly worked on the abolition of the FGC. Elderly, male, and female key informants were purposively chosen from the community to gather data on the cultural values underlying FGC and the community's reactions to government interventions to abolish it. The key informant interviews took between thirty minutes with community members and ninety minutes with an expert from the office of WCYA.

Focus group discussion

We also used focus group discussions (FGDs) to triangulate the data from key informant interviews. To look at the concerns of different individuals and even explore their common understanding of some issues, we organized two FGDs. In the first FGD, there were eight experts (four men and four women) from concerned offices: two from the office of WYCA, two from the Culture and Tourism office, one from the Education Office, a schoolteacher, and two from the district Police. The participants of the second FGD were nine married women who had experienced FGC as recipients and mothers. This method helped to access data from individuals as members of a group rather than simply as individuals. The discussions, debates, and consensus were all important because by bringing together different individuals who have a common

background; FGD plays an essential role in building meaningful data from common societal issues and confrontations.

Data collection tools and analysis

For both key informant interviews and FGDs, we used semi-structured questions that were few in number and open-ended in nature, with the objective of eliciting views and opinions from the participants. These questions were set with the intention of guiding the informants rather than restricting them to answering what was just asked. All the interviews and FGDs were conducted in the local language, which we, the researchers, are native speakers of, and the principal investigator is familiar with the research site. We transcribed the data into the original language and translated it into English. We coded, described, categorized, and interpreted the data qualitatively.

Ethical consideration

Before conducting the study, ethical clearance was obtained from the Research and Ethical Review Board (RERB) of the College of Social Sciences and Humanities of the hosting university (the name of the university is temporarily remained confidential for article reviewers). After reviewing the proposal and specifically the participants ‘consent form to be signed, the RERB fully authorized the conduct of the research. The research did not involve minors; and that the study was conducted by implementing all human handling during the data collection. All the participants were informed about the purpose of the study and provided their consent in written form to participate in the research. The written consents were for the consumption of the researchers and RERB. Finally, the findings of the study were reported in an aggregate fashion, codes were used to quote participants and no personal names are mentioned in this article.

Result

Approaches to end FGC and community resistance in Dawo district

The micro-level efforts to end FGC are direct reflections of the national policies, rules, and regulations. The major macro-level approaches are the human rights approach, the legal approach, the health risk approach, training health workers as change agents, and training and converting the circumcisers.

As indicated in the introduction, Ethiopia introduced a penal code banning FGC (Ethiopian Criminal Code Art. 565 and 566). In 2013, the country introduced a National Strategy and Action Plan on Harmful Traditional Practices against Women and Children, which has been followed up by another strategy known as the Social Norm Change Communication Strategy. The strategy to end HTP is based on the three-pillar approaches of prevention, provision, and protection [13]. The country set up a national alliance to terminate child marriage and FGC that is led by the MoWCY, in which other sectorial ministers, national associations, and faith-based, international, and local civil society organizations have collaborated on the implementation of the national strategy. In addition, the Ethiopian government has restated its plan to stop FGC and child marriage by 2025 [11].

In January 2017, the Ministry of Health banned the medicalization of FGC in all public and private medical facilities, and any breach of this ban will be subject to legal action. The restriction goes hand-in-hand with the criminalization of FGC under the Criminal Code of 2005 [14, 11]. As an extension of the criminalization of the act, the district police office has a focal person who works to protect the human rights of women and children. The focal person is in charge of handling cases such as early marriage and/or abduction, rape, and violence against women, including FGC.

The MoWCYA at the federal level has offices at regional, zonal, and district levels to implement national policies and strategies to end FGC and to ensure gender equality (The Ethiopian federal state has regional, zonal, district, and *kebele* levels of administration) [11]. There is a district office for WYCA, designed to oversee and protect the rights and well-being of women and children. The FGC is one of the central focuses of this office. The Office of Culture and Tourism is also in charge of promoting cultural elements and fighting against "harmful traditional practices." The schools are also responsible for teaching the young generation to develop all-rounded personalities. They attempt to create awareness about the adverse effects of practices such as FGC and early marriage. These offices and institutions work in cooperation and even as a team against the FGC [36, 37].

According to Boyden et al. [16], the national FGC network that was aimed at fighting FGC was established in Ethiopia in 2002 under the initiative of Norwegian Church Aid. The network was officially launched in 2010 and has 46 members from government, NGOs, faith-based and civil society organizations, and UN agencies (notably UNFPA, UNICEF, and WHO). Ethiopia also introduced a health extension package to reach villages and households through health extension workers. Among others, the health extension workers focus on creating awareness to end harmful traditional practices (HTPs), including FGC.

The persisting of FGC in Dawo

Our data from the primary sources [38,39] show that the community is well-informed about the criminalization, the negative health consequences, and the related violations of human rights of the practice of FGC. However, mere knowledge of the issue has not deterred the community from practicing FGC. The primary data from the field imply four explanations for the persistence of this practice. Primarily, the community gives precedence to the cultural values of FGC over its negative

health effects and its legal implications. Secondly, the community has designed a mechanism to escape the potential legal prosecution that could result from FGC. Thirdly, the community generally undermines the health effects of FGC by appealing to their lived experiences. Fourthly, the community also disregards the fines that could result from FGC. On the other hand, the issues of individual rights, bodily integrity, and sexual pleasure are not a point of attention for the community. The issue of individual rights has no space in this community because it is collectivistic, focusing on 'we' consciousness, collective identity, and emotional dependence rather than the opposites. The norm does not also allow the discussion of the second issue—sexual integrity—in public. In the following sections, we focus on the explanation of these issues.

Cultural values vis-à-vis approaches to end FGC

The community provides multi-layered cultural reasons for practicing the FGC that are intended to challenge the government's intervention efforts to stop it. The main cultural explanations they provide are sexual modesty, preserving pre-marital virginity, and developing accepted feminine behavior that ensures the marriageability of the girl. The community uses the compound term "*dhaqnaqabaa"* or "*huuba irraa fuudhuu"* to refer to both FGC and male circumcision. The term *dhaqna* refers to the body, and *qabaa*, from *qabuu*, to 'catch'. Literally, it means 'catch the body to circumcise. According to our participants [40, 41, 42] and FGDs [37] a woman who has not undergone FGC is clean neither physically nor ritually.

This fact is further cleared by the phrase "*huuba irraa fuudhuu*", which means to "remove something unwanted." The term "*huuba*" means 'unwanted', or 'smut'; *irraa fuudhuu,* means "to pick" or "remove something from a surface." Together the two words mean "eliminate a chip or something malignant or unwanted." The phrase "*huuba irraa fuudhuu"* also implies that female genital cutting is as important as male circumcision [43]. The expression shows that there is

something external and, therefore, unwanted on the genitalia, and due to this, should be removed. For the community, FGC is the surgical removal of the phallic and "unnecessary" or "unwanted" part of the genitalia, the clitoris, to make her a real woman, both sexually and physically because the phallic is for men, and therefore, should be removed from the females.

Beyond the physical concern, the surgery entails a behavioral change that ensures femininity in terms of gender. The ultimate goal of FGC is to control women's sexual drive and ensure sexual decency, premarital virginity, and marriageability. There is an assumption that girls who have not undergone FGC are hypersexual, unclean, and indulging. As a result, our data from primary sources unanimously showed that girls who have not undergone FGC are not esteemed and wanted for marriage. This is so because they still have the erectile part of their genitals (the clitoris), have not yet attained femaleness physically, and are not also properly feminine in their gender.

The community uses different sayings to ridicule women who have not yet undergone genital cutting. Referring to the physical presence of the clitoris, they say *ishiin eeboo baatti, dankaraa baattee deemti*, 'she carries a spear'. Focusing on the sexual behavior and gender identity of the uncircumcised girl, the community often uses such sayings as *ishiin farda*,'she becomes disorderly (like a horse);' *safuu dhabdi*, 'she is unethical;' *ija re'ee nyaatti*, 'she becomes shameless;' *abbaa manaa jala hin bultu*, 'she cannot remain with one husband'; *dhaabbattee hafti*, 'she will remain a bachelor'[37, 44] Similarly, UNICEF [6] shows how deviating from this norm has social costs, including ostracization, in which girls are labeled as being 'unclean', 'clumsy', 'unfit' for domestic work, or 'having uncontrolled sexuality', and thus unmarriageable.

Men do not choose such girls for marriage. If they married, their marriage would not be successful, mainly because of the fear of hyper-sexuality and untidiness. One of our participants, a woman, tried to substantiate this view by narrating the story of a certain unsuccessful marriage. She knew an uncircumcised woman who married and divorced three times. According to this informant,

the divorcee did not have a stable marital life mainly because she had not undergone genital cutting and because she challenged those men who married her [40]. We, the researchers, did not confirm the story and do not know the real reasons behind the frequent divorce of the woman in focus. However, this informant, who is a pro-FGC woman directly, associated a stable marital life with FGC and strongly recommends it as the best therapy to ensure a successful marital life for a woman. As she stated, all her daughters have undergone the FGC.

Within the prevailing patriarchal cultural framework, women, as such, mothers and grandmothers, are the prime promoters of FGC because they are given the irreplaceable role of socializing and shaping, including the sexual behavior of girls, and one of the instruments for this is FGC. If a girl behaves in a customarily wrong way, the problem is soon attributed to the way she has been brought up. To express this fact, they use the common expression *isheen guddisa badde*, 'she was badly brought up.' If the misconduct is related to sexual activities, the blame becomes more severe and goes to the mother or the grandmother who brought her up.

A mother whose daughter has not undergone FGC often receives shaming remarks from others. Sayings such as *Intalli kee saafeela kan dhaabbattee hafte,* 'You are the mother of the uncircumcised, therefore, unmarried girl'. It is so shameful and dishonoring to be insulted in this way. In another saying, *haadha ilaali intala fuudhi*, 'see the mother before marrying her daughter', similar to the saying 'all daughters grow up to be like their mothers' [39, 44], members of the community convey the strong message to families that they have to cut the genitals of their daughters if they want to have their daughters to have a sustained marriage.

In substantiating this view, a father-informant whose wife organized a genital-cutting ceremony for their daughters without his consent narrated the following story of his family. Our informant has two young daughters. He claims that he is aware of the negative impacts of FGC because he completed a formal education of grade 10 while his wife, who has no formal education, is

a proponent of the practice and believes that FGC is mandatory and necessary for the cleanliness of women and cultural conformity. According to him, she believes it is her responsibility to raise her daughters and mold them into ideal female behavior. If her daughters remain uncut, she would be responsible for the disgraceful state of the girls. Thus, the wife of this man organized a secret genital-cutting program for her young daughters without her husband’s knowledge and any ceremony. She assigned her sister-in-law as a *jaalaa* (sponsor) to keep the case confidential [46].

The father recognized lately that his daughters had undergone FGC. He noticed the girls receiving wound treatment while returning home from his daily farming activities. He strongly indicated that for a mother, letting her daughter remain with her clitoris is an incurable and lifelong psychological and moral failure. He said that *rakkoon dubartoota bira jira. Ani kanan dhaqna qabadhe maalan ta’e jedhu. Ani qofti mucaa koo dhaqna qabsiisuu dhiisee maalifan kan kolfaa ta’a; mucaa kootti kofalchiisa jedhu.*

The problem is with the mothers. They say though I have already undergone FGC, what happened to me? Why do I make my daughter and myself mocked at by others leaving her uncut?’ [46]. We were able to talk to the mother who organized a secret session to cut her daughters’ genitals. She proudly explained how she was successful and admitted that the report from her husband was true [47]. Related to this issue, El-Dirani et al. [26] show the nexus between parental education and values for FGC in Africa. These authors argued maternal education matters, and the more educated mothers are the less they practice FGC on their daughters, and vice versa. This is confirmed by the participating school teachers who have daughters and have not performed FGC and would not also perform it. Three female teachers who have already had daughters reported that they have not exposed their daughters to FGC and would not do so. The reaming two female teachers argued that in principle they are against the practice; however they are not sure what would happen when they will have daughters as the issue of FGC entails the interventions of several stokeholds in the extended

family. One of the male school teachers admitted that his elder daughter has already undergone FGC under the pressure of his mother and the junior ones have not undergone as he and his wife strongly resisted the pressure [48] The second male participating teacher who has a young daughter of two years said that he would not perform any FGC [49].

Coupled with this, the prevailing cultural norms put pressure on girls to have their genitals cut. Our data indicated that peer groups and friends harass, tease, and laugh at girls who have not undergone genital cutting [50]. One of our participants, who claim to be aware of the negative consequences of FGC, narrated to us how he finally submitted to the firm demand of his only daughter to have her genitals cut. The father says that when she was eleven years old, she strongly demanded to undergo genital cutting; otherwise, she would stop going to school and escape somewhere. The father says that he was helpless to reject this demand from his daughter, for the denial might lead to a worst-case scenario, i.e., the disappearance of their daughter. In the meantime, he also believed that there was a hidden hand of his wife behind the pressure that their daughter was exerting. His wife did not openly oppose his position but agreed with her daughter and designed the way to achieve the final goal of cutting the genitalia [51].

Withstanding the criminalization of FGC

Despite the campaign against the FGC by the government, the practice is still widespread, and the legal actions against it by the judiciary are closest to nil. This is mainly because the community withstands the potential legal actions either by paying the imposed fine or by hiding the practice from legal or concerned bodies to escape being prosecuted [38, 44, 52, 53]. According to our participants, several factors contribute to this effect. Primarily, the law that forbids FGC lacks any deterrent effect. The law was enacted some years ago, and given the fast devaluation of the Ethiopian currency, a fine of five hundred Birr (as stated in Article 565 of the Ethiopian Criminal Code) has no preventive effect. One of our participants expressed the amount of the fine, saying, *dhibbi shan gatii indaanqoo*

tokkooti; eenyu illee adabbii qarshii dhibba shaniif jedhee dhaqnagabaa hindhiisu. 'Five hundred Birr is just the price of a rooster; this penalty never deters anyone from practicing FGC [46]. Regardless of its deterrent effect, our data indicates that the police, in cooperation with the Office of WYCA, brought a few cases of FGC before the district court and penalized the actors, including parents, sponsors, and circumcisers, as per the Ethiopian Criminal Code Article 565. Yet, parents are always ready to cover the penalties instead of refraining from the practice because the fine is so meager and one that can easily be afforded [37, 42].

Concealing FGC

Alternatively, at best, parents hide FGC from legal personnel to escape prosecution. There are several ways to do this. Primarily, the local procedures have undergone a dramatic change in terms of the setting of the practice. The changes are in terms of the age at which girls get their genitals cut, the practitioners who do the surgery, the selection of the sponsors, the circumcision rituals, and the publicity of the practice. Data from interviews and FGDs indicated that there is no specific age at which girls get their genitals cut. It varies greatly based on parents' interests and readiness, convenient time to make the practice confidential, and external factors such as government intervention, and so on.

The community maintains the confidentiality of the practice by adjusting its timing, season, and occasion. Nowadays, parents subject their daughters to FGC at a young age, a few days or months after delivery, when it is less likely for anyone other than immediate family members, the sponsor, and the practitioner to know about the procedure. This enables parents to maintain maximum confidentiality. Our informant from the office of WYCA complained that cutting baby girls' genitalia at an early age is one of the challenges to stopping FGC. EUAA [14]

shows that the age of genital cutting for more than 70% of Ethiopian girls is below 10 years, and about 49% have undergone genital cutting before they celebrate their 5th year. Parents pretend that they do not perform FGC. In this regard, Camilotti [34] shows how individuals in rural Ethiopia, particularly older ones, are in favor of FGC while concealing their true support for it.

Secondly, parents arrange *jaalaa* (sponsor) from the same family or close relatives to keep the practice private. Traditionally, sponsors come from outside the family because it is one way of establishing fictive kinship ties. Under the current scenario, priority is given to performing genital cutting in favor of the required cultural conformity rather than establishing kinship ties [45].

The third way of hiding FGC is by confusing it with other religious or non-religious festivities. This is common during the week after the Ethiopian Easter in April or early May every year. This is the time when Ethiopian Orthodox Church followers, who are the majority in the area, end the two-month-long fasting period. This is also the most joyful religious and cultural occasion when people visit and invite each other. It is the best time to confuse FGC with the festivities of the season. Similarly, other non-religious ceremonies and occasions, like marriage ceremonies also serve to confuse the FGC [37].

The fourth way to keep FGC confidential is by confusing it with male circumcision. This is easy when the parents have boys and girls who are ready for circumcision. Parents make the necessary preparations officially to circumcise boys. Covertly, the same ceremony is used for the FGCs of their daughters. In some cases, parents use a male circumciser to do FGC. The use of male circumcisers for FGC is indeed a new phenomenon [42].

When such confusion happens, the circumcised boys appear at the front bed, where visitors can see, talk, bless, and encourage them. Neighbors and relatives pay a visit to the circumcised to wish him a speedy recovery from the wound. They usually say, *"Madaan madaa*

saree siif haa ta'u," meaning, "Let your wound be a dog's wound." A dog's wound heals quickly because the dog keeps it clean. Girls stay in the backyards to be out of sight of the visitors. The family pretends that the entire ceremony is held for the circumcision of the boys. The fifth alternative way of hiding FGC is by taking girls to another place in the name of visiting their relatives. This is more common for urban dwellers who are not confident enough to cut the genitals of their girls for fear of being prosecuted or being blamed by their friends. In this case, the confidentiality of the cutting can be easily maintained from the outside, as the girls are guests there [42].

Gibson et al. [35] also report that in Senegal, the practice of FGC was concealed to avoid being prosecuted. Under the threat of legal measures, people have two options: either to stop practicing it or to continue doing it secretly. It is less likely that the fear of prosecution can lead to the abandonment of FGC; rather, individuals may opt for other alternatives to decrease or escape the risk of being prosecuted.

Our finding also shows that FGC has the paradoxical effect of concealing and disclosing at the same time. Parents try to hide the act from the police and the Office of Women, Youth, and Children Affairs to escape legal prosecution. On the contrary, they also want the exercise to be known to the public at a certain point to secure cultural approval [51, 52, 53, 54,55, 56]. The parents want their friends and neighbors to witness the practice so that any potential teasing or mockery by others could be averted. With this, mothers also make themselves free from being blamed for letting their daughters go with an "unwanted"[57, 58, 59].

Lack of Collaboration

The more solid challenge to stopping FGC is the lack of cooperation between members of the community and the concerned offices, notably the police and the Office of WYCA, the two offices that primarily work on the abolishment of the practice of FGC. However, they rarely

access information on any planned and/or practiced FGC in the district. This is because the community considers FGC as a normal practice that does not demand the intervention of the police or any other government body. For the larger community, FGC cannot be any good reason to expose one's neighbors, friends, or relatives to any legal prosecution. One of our participants stated that *warri mucaa isaanii yoo dhaqna qabatan, miidhaas ta'ee faayidaan kanuma isaaniti. Isaan caalaa mucaa isaanii kan jaalatu eenyuu? Maaltu poolisii bira nama geessa?* 'If the parents do FGC for their daughter, the advantages and disadvantages will be theirs. Who else can care for a child more than the parents? What reason can take one to the police?'[46]. In the absence of any information from the community, therefore, it is less likely that the government body will intervene to tackle the FGC.

In addition, data from the police office indicates some challenges to enforcing the law to prohibit FGC. These include logistics problems, poor infrastructure, a lack of budget, and other facilities. For instance, a shortage of transportation facilities denies the police to either intervene to abort some planned FGC or react to legal measures in time [37, 44, 53, 54]. There is also a paradox in the execution of the law against FGC. Usually, when the police identify a case of FGC, they take the injured girls to health centers for medical treatment and arrest the parents for committing the act. The questions are, "Who must take care of the victims if the parents are under police custody? Who should afford the required expenses for medication? Where the victims should be accommodated? What transportation facilities are available, and who should provide the services for the girls who have already been injured and cannot walk on foot?" Since the office of the police does not have the logistics and budget for these services, they usually become reluctant to imprison or impeach the parents.

A participant from the police office narrated one instance as follows: in May 2019, the police found four girls who had undergone genital cutting. The parents were arrested, and the

girls were taken to the health center for medical checkups and treatment of their wounds. The health center provides only outpatient services. The police staff individually contributed to cover the expenses for medication, as there is no government budget for this kind of service. Finally, the police had to spot where to keep the girls. Because they needed all kinds of help and supports like care for their wounds, what to eat and drink, and one who could look after them since their parents were already detained. Finally, the police decided to free the parents and allowed them to take care of their daughters [37].

Discussion

The local phrase *dhaqna-qabaa* stands for both male and female circumcision. According to Llamas [9], the comparison of male circumcision and FGC is a tendency to normalize the latter. Our findings revealed that the public perceives circumcision as a normal surgical procedure to remove something unnecessary both from male and female genitalia, respectively. The phrase *huuba irraa fuudhuu* in particular suggests that both male circumcision and FGC are important since they remove the unwanted part of the genitals through an easy procedure. Thus, the

For the community, FGC is valued for physical cleanliness, sexual decency, gender construction, and marriageability. These findings partly corroborate IPPF [2], which shows that some of the justifications behind FGC include the preservation of virginity, ensuring fertility, rite of passage, family honor, marriageability, hygiene and cleanliness, infant survival, increasing sexual pleasure for the male, and religion. Our findings, however, did not show any explanation related to religion, infant survival, fertility, or male sexual pleasure for practicing FGC in the research area.

This study showed that the overall cultural framework that favors FGC is patriarchy. This is more vivid in terms of what Morrison [22] labeled as the "clitoral economy." This concept

depicts the balance of the female body (clitoris) and the male body (penis). The study revealed that the culture bestows the clitoris with special symbolic power over the female body. The community refers to the clitoris as phallic which challenges the hegemonic, phallic economy of men. Since the culture tends to reserve something phallic for males. FGC is the best therapy to remove it. This is typical patriarchy, which works to control women's sexuality and many more to the benefit of males.

Generally, patriarchy works behind the scenes and men are not at all at the forefront of promoting FGC. That means women are the prime promoters of the practice for they are the products of the longstanding patriarchal values. Therefore, to understand the value of FGC, it is important to consider the role of women and their perceptions of ideal women's bodies, beautifications, and body modifications within the framework of patriarchal values. Women want to shape the sexual and moral behavior of their daughters so that they will meet the ideal standard of womanhood. Our finding corroborates Ahan's [23] view, which explicitly declares that the causal explanation behind the survival of FGC is not only patriarchy which provides the guiding principle behind the scenes but also some other products of the patriarchal culture.

The study showed that the participating mothers strongly supported FGC for their daughters, even when their husbands were reluctant to do it. This is because what matters is not the position of individual husbands but rather the wider patriarchal cultural setting. The findings further indicated that the cultural framework in which they act—patriarchy—imposes mothers' absolute responsibilities in shaping the behavior of their daughters, including their sexual behavior. This finding confirms UNICEF [6], which shows that the prevalence of FGC depends more on some background characteristics. Less educated girls and women in rural areas are more exposed to FGC. The finding is also relevant to Abebe et al. [7], Oljira et al. [28], and Geremew et al. [27] who show the nexus between maternal education and the FGC of their daughters. The

findings of this study showed that resistance comes from uneducated mothers, at least, while the husbands who claimed to be aware of the negative consequences of FGC are ready to deviate from the norm. In this regard, the education of the mothers determines their position.

The government attempted to bring FGC to an end through both health and legislative approaches. The health and psychological effects of FGC emphasize women's inalienable rights, especially bodily integrity and sexual pleasure [32]. Our findings showed that the local community is aware of the perceived negative health effects of FGC, mainly the risk of infection and childbirth complications. The sexual integrity approach has not been explicit in the local efforts to stop FGC. The custom does not allow the discussion of sexual pleasure as a public agenda (as a point of justification) to end FGC.

However, the community does not accept the health approach at face value. Particularly, older women strongly challenge and disqualify the scientific explanations and justifications given regarding this point. They often refer to their lived experiences to override the stated negative effects of the practice. This finding also corroborates the studies by Backlund and Blomqvist [24] and Emire [25], who discuss how older women act as active agents to reinforce patriarchal traditions by influencing younger girls to abide by the traditions they have been through.

Our finding also supports the view of Brown et al. [29], who present that the bodily and sexual integrity approach is challenged because sexual enjoyment is also cultural. Cultural values remained strong enough to withstand the theoretical orientation and awareness-creation of various agents. The issue of sexual pleasure is not part of the discussion either to justify or resist FGC. Similarly, the human rights approach is neither fully understood by the public nor part of the public discourse. The overall practices suggest the precedence of cultural values over private or individual rights.

The Ethiopian Criminal Code has labeled FGC as an illegal act. Some news of prosecution and the threat of trial are also public. Yet, the commandment has borne no fruit; rather, empirical instances show that the practice of genital cutting has persisted. FGC is illegal only from a legal perspective; it does not win public acceptance and recognition in practice. The legislative approach has faced serious challenges and caused unintended adverse effects for the act inviting less experienced female or male practitioners to do the cutting and for the parents to refrain from seeking medical treatment for the recipient's medical complications. This finding substantiates Nakamura et al. [1], who show how parents practice FGC underground and abstain from seeking health care for any complications that may result from the surgery. Similarly, medical personnel also fear to treat the victims who received FGC and abstain from getting involved in it.

Brown et al. [29] have already questioned the effectiveness of the legislative approach in changing people's behaviors to end FGC. Gudeta et al. [18] also show the prevalence of female genital cutting in many countries where laws prohibit it because the practice is conducted secretly. Our finding supports these views in that the legislative approach lacks deterrent effects. Firstly, only a few individuals were prosecuted for cutting girls' genitalia. This seems common in other parts of the world. For instance, IPPF [2] shows despite the presence of national legislations that prohibit FGC in several African and Asian countries, the practice remains common and, only a few cases of FGC are taken to court for prosecution. Abebe et al. [7] who studied FGC in the northern and northeastern parts of Ethiopia reported that many such cases remained unreported for the reason that the community performs it secretly in the distance and remote areas where the judiciary might not have access to information or secure evidence to prosecute the offenders and enforce the law. Secondly, this study indicates that the deterrent effect of these legal measures is under question, for the fine is so meager that everyone can easily afford it. Mothers are ready to

pay whatever the price could be rather than being ashamed by letting their daughters remain uncut. Moreover, the very idea of banning a cultural practice through a legislative approach has its own challenge. As Muteshi and Sass [32] state, this way of attempting to end a cultural practice causes a feeling of top-down imposition and faces community resistance.

Finally, the findings suggest that extensive public awareness creation, working with the local elders (men and women), and educating girls about its multi-faceted implications are more viable ways of ending the practice of FGC.

Acknowledgement

We would like to express our special thanks and gratitude to the participants who provided us with valuable data to write this article. We also owe thanks to the local government officials in the district for cooperating with us to conduct this research.

References

1. Nakamura K, Miyachi K, Miyawaki Y, Toda M. *Female genital mutilation/cutting: global zero tolerance policy and diverse responses from African and Asian local communities.* Springer Nature. 2023.
2. IPPF. Female genital mutilation (FGM). Briefing Paper. vardgivare.regionorebrolan.se. 2014.
3. CSA. Population projections for Ethiopia 2007–2037. Federal Democratic Republic of Ethiopia, Population Census Commission Addis Ababa. 2013.
4. Central Statistical Agency (CSA). 'The 2007 Population and Housing Census a preliminary Result'. Retrieved May 10, 2020 from http://www.csa.gov.et/census-report/complete-report/census-2007. 2007.

5. Gemechu DG, Mekuria ZM. Huuba irraa Fuudhuu: Female Genital Cutting among the Oromoo, in Dawo District, Ethiopia. *Gadaa Journal*. 2021 Jun 30;4(2):36-58.
6. UNICEF. A profile of female genital mutilation in Ethiopia. UNICEF Ethiopia, February. 2020.
7. Abebe S, Dessalegn M, Hailu Y, Makonnen M. Prevalence and barriers to ending female genital cutting: the case of Afar and Amhara regions of Ethiopia. *International journal of environmental research and public health*. 2020 Nov;17(21):7960.
8. Waigwa S, Doos L, Bradbury-Jones C, Taylor J. Effectiveness of health education as an intervention designed to prevent female genital mutilation/cutting (FGM/C): a systematic review. *Reproductive health*. 2018 Dec;15:1-4.
9. Llamas J. Female circumcision: The history, the current prevalence and the approach to a patient. University of Virginia Medical School Working Paper. 2017 Apr:1-8.
10. Central Statistical Agency (CSA) [Ethiopia] and ICF. *Ethiopia Demographic and Health Survey 2016*. Addis Ababa, Ethiopia, and Rockville, Maryland, USA: CSA and ICF. 2016.
11. Federal Democratic Republic of Ethiopia. Synthesis Report on the Implementation of the Beijing Declaration and Platform for Action (Beijing +25). 2019.
12. Federal Democratic Republic of Ethiopia (FDRE). (2005). The Revised Criminal Code Proclamation No. The Proclamation No. 414/2004 *Federal Negarit Gazetta*, 2005-05-09, extraordinary issue, Addis Ababa. 2005.
13. Federal Democratic Republic of Ethiopia, Ministry of Women, Children and Youth (MoWCYA). National Strategy and Action Plan on Harmful Traditional Practices (HTPs) against Women and Children in Ethiopia. Addis Ababa. 2013.

14. European Union Agency for Asylum (EUAA). Female Genital Mutilation/Cutting in Ethiopia Country of Origin Information Report. 2022
15. Sally Effie O. Why does female genital mutilation persist? Examining the failed criminalization strategies in Africa and Canada. 2015
16. Boyden J, Pankhurst A, Tafere Y. Harmful traditional practices and child protection: Contested understandings and practices of female child marriage and circumcision in Ethiopia. Young Lives; 2013.
17. UNICEF. The dynamics of social change towards the abandonment of female genital mutilation/cutting in five African countries. Innocenti Insight. 2010;5:6-23.
18. Gudeta TA, Regassa TM, Gamtessa LC. Female genital mutilation: prevalence, associated factors and health consequences among reproductive age group women in Keffa Zone, Southwest, Ethiopia. *Reproductive Health.* 2022 Mar 5;19(1):60.
19. AIDOS – Associazione italiana donne per lo sviluppo. Female Genital Mutilation/cutting. A quick guide for Media. 2016.
20. World Health Organization. Understanding and addressing violence against women: Intimate partner violence. World Health Organization; 2012.
21. Diop MK, Stewart P, Herr K, Kani-Diop M. A Black African feminist theory to examine female genital mutilation (FGM) within African immigrant families in the United States. Inunpublished contribution delivered at the National Council on Family Relations Annual Theory Construction and Research Methodology Workshop 2017 Nov (pp. 14-15).
22. Morrison BS. Feminist theory and the practice of female genital mutilation (FGM). 2008;26:2-0.
23. Ahan, Farnoosh Rezaee. Theories on Female Genital Mutilation. Appsala Universitet. available at: http://ssrn.com/abstract=2250346. 2012.

24. Backlund H, Blomqvist G. Protecting the girl child or upholding patriarchy. A case study of child marriage in the cultural and legal context of Tanzania. Lund University: SIDA. 2014 Jun.

25. Emirie G. Early marriage and its effects on girls' education in rural Ethiopia: The case of Mecha woreda in West Gojjam, north-western Ethiopia. 2005.

26. El-Dirani Z, Farouki L, Akl C, Ali U, Akik C, McCall SJ. Factors associated with female genital mutilation: a systematic review and synthesis of national, regional and community-based studies. *BMJ sexual & reproductive health*. 2022 Jul 1;48(3):169-78.

27. Geremew TT, Azage M, Mengesha EW. Hotspots of female genital mutilation/cutting and associated factors among girls in Ethiopia: a spatial and multilevel analysis. *BMC public health*. 2021 Dec;21:1-8.

28. Oljira T, Assefa N, Dessie Y. Female genital mutilation among mothers and daughters in Harar, eastern Ethiopia. *International Journal of Gynecology & Obstetrics*. 2016 Dec 1;135(3):304-9.

29. Brown K, Beecham D, Barrett H. The applicability of behaviour change in intervention programmes targeted at ending female genital mutilation in the EU: integrating social cognitive and community level approaches. *Obstetrics and gynecology international*. 2013;2013(1):324362.

30. Johansen RE. Experiencing sex in exile–can genitals chenge their gender? On conceptions and experiences related to female genital cutting among Somalis in Norway. I: Hernlund, Ylva K. & Bettina K. Shell-Duncan (eds)(2007) Transcultural bodies: Female genital cutting in global context, Rutgers University Press; 2007.

31. Odukogbe AT, Afolabi BB, Bello OO, Adeyanju AS. Female genital mutilation/cutting in Africa. *Translational andrology and urology*. 2017 Apr;6(2):138.

32. Muteshi J, Sass J. Female genital mutilation in Africa: an analysis of current abandonment approaches. Nairobi: PATh. 2005.
33. Andarge MY. The difficulties of ending female genital mutilation (FGM): case of Afar pastoralist communities in Ethiopia. Retrieved from www ohchr org/Documents/Issues/Women/WRGS/FGM/NGOs/ActionFor. 2014 Dec 15.
34. Camilotti G. Interventions to stop female genital cutting and the evolution of the custom: Evidence on age at cutting in Senegal. *Journal of African Economies.* 2016 Jan 1;25 (1):133-58.
35. Gibson MA, Gurmu E, Cobo B, Rueda MM, Scott IM. Indirect questioning method reveals hidden support for female genital cutting in South Central Ethiopia. *PloS one*. 2018 May 2;13 (5):e0193985.
36. FGD by experts; Busa, March 8, 2020
37. FGD by married women, Kalacho, March 11, 2020
38. Interview with district police focal person, age 38; Busa, March 6, 2020
39. Interview with a male elder, age 60; Busa, March 5, 2020;
40. Interview with a female key informant, age 48; Busa, March 10, 2020
41. Interview with a female key informant, age 58; Busa, March 7, 2020;
42. Interview with a female schoolteacher, age 38, Busa, March 9, 2020
43. The surgery is mandatory for males too, since he who has not undergone it is said to be unclean. A male person who is not circumcised is insulted with a derogatory word ‘uncircumcised’ or ‘one whose prepuce is still intact.’
44. Interview with district WCYA expert, female age 40; Busa, March 5, 2020.
45. A sponsor is someone who sits behind the circumcised person and covers his or her eyes during the procedure. He or she tightly grips the circumcised to prevent any movement in

response to the pain that may result during the cutting process. Hereafter, a sponsor takes partial responsibility for bringing up the circumcised.

46. Interview with a male key informant, age 45; Busa, March 7, 2020
47. Interview with a female key informant, age 43; Bashi, March 7, 2020
48. Interview with male schoolteacher, age 42, Busa, March 9, 2020
49. Interview with male schoolteacher, age 52, Busa, March 9, 2020
50. Interview with a female schoolteacher, age 34; Busa, March 6, 2020
51. Interview with a male key informant, age 42; Busa, March 9, 2020 (he had formal education, rural resident but government employee, while his wife has no formal education).
52. Interview with a female schoolteacher, age 46; Busa, March 6, 2020
53. Interview with office WCYA expert, male age 38; Busa, March 5, 2020.
54. Interview with a female district WCYA expert, age 32; Busa, March 5, 2020
55. Interview with female schoolteacher, age 28, Busa , March 10, 2020
56. Interview with a female schoolteacher, age 29, Busa, March 9, 2020
57. Interview with a female key informant, age 63; Busa, March 8, 2020;
58. Interview with a female key informant, age 37; Busa, March 8, 2020 (education office)
59. Interview with a male key informant, age 33; Busa, March 9, 2020 (culture and tourism office)
