# Supplementary material for: Forbidden, yet common: Female genital cutting among the Oromo in central Ethiopia
Source: PLoS One. 2024 Dec 17;19(12):e0315566. doi: 10.1371/journal.pone.0315566 (PMC11651596; doi:10.1371/journal.pone.0315566)
Supplement: Attachment — Submitted filename: Response to reviewers comment, final.docx [file pone.0315566.s003.docx]

**Responses to comments**

**A. Editor’s comment**

1. **The description of the study design is not clear.**

**Response**:

The editor’s comment was addressed in the ‘Methods and Materials’ section. We have added a new sub section ‘Research Design’ (*page: 11, line 1-2*).

2. **There is a mention of secondary data being used, however, it is not clear how this data was retrieved, the source, how it was accessed, etc.**

**Response:**

How the secondary data in the manuscript were used was also addressed in the ‘Methods and Materials’ section of the manuscript *(page: 11, line 2, 8, 11 and 13*).

3. **There should be a clear description of the link between primary and secondary data.**

**Response**:

The link between the primary and secondary data was described in the ‘Methods and Materials’ section of the manuscript as well (*page: 11, line 12 to 13*).

**B. Reviewer 1**

1. **Consider adding a paragraph at the end of the manuscript that provides recommendations or conclusions based on the study's findings**.

**Response**:

We have added ‘conclusion and recommendations sub section’ and paragraphs at the end of the manuscript in which we incorporated the comments of Reviewer 1 related to the roles of religion in FGC, the regional variations of FGC practices in Ethiopia, and recommendations that hint at ways forward about the topic of the manuscript in the research site.

2. **While the authors effectively concluded that patriarchy and male dominance underpin FGC, it's essential to acknowledge that patriarchy is deeply entrenched in societies. Understanding and addressing the factors that strengthen or weaken the patriarchal system can influence the practice of FGC.**

**Response**:

A recommendation is added in ‘Conclusion and recommendations’ (*page 29 line, 22 to page 30 line 1*)

3. **The paper could benefit from discussing the role of religion and religious leaders in FGC. DO you have any reference or have any finding which explains the role of church/religious leaders?**

**Responses**: The role of religion and religious leaders in FGC is discussed ‘in the discussion section’. However, religion and religious leaders have no role in advancing or terminating this practice in the research site (*pages: 25 line 20 to 23*).

4. **Consider comparing the findings with quantitative data from the available studies.**

**Response**:

The different quantitative studies conducted on FGC at national, regional and specific research sites have been presented in the manuscript (*page 3, line 12 to 15 and 22 to 23; page 4 line 1 to 3; page 21 line 20 to 21; page 28 line 2*)

5. **One of the key recommendations from your study is to strengthen the law regarding Female Genital Cutting (FGC). This can be achieved by making the law more powerful and effective as a deterrent to those who promote or practice FGC. It can be achieved by increasing fines or punishments, improving law enforcement through active case finding, and providing appropriate care and shelter to FGC victims once they are rescued.**

**Response**:

These issues are incorporated in the ‘conclusion and recommendations’ subsection of the manuscript (*page 30 line 1 to 6*).

6. **Combating FGC requires allocating resources for advocacy, policy change, enforcement, awareness-raising, and engagement of civil society, parents, girls opposing FGC, and religious leaders.**

**Response**:

These points appeared in the ‘Conclusion and recommendations’ section (*page 30 line 4 to 6*)

7. **Giving examples from regions where FGC has been eradicated and highlighting successful strategies would be beneficial.**

**Response**:

Although there is no region in Ethiopia that is free from FGC, we recommended that Oromia, the region where our study site is situated, has to draw a lesson from those regions where the practice is prevalent but to a lesser degree (*page 6 to 8*).

8. **“This study showed that the overall cultural framework that favors FGC is patriarchy.” How do you conclude this?**

**Response**:

In this manuscript, we have argued that the social structure of the people in the research site is patriarchal and how the social structure has taken precedence over the legal and women’s rights.

9. **“The community refers to the clitoris as phallic which challenges the hegemonic, phallic economy of men. Since the culture tends to reserve something phallic for males, FGC is the best therapy to remove it.” It is not clear; do you want to say: the nature preserves/ reserves something phallic for females?**

**Response**:

The sentence is rewritten for clarity (*page 26 line 6 to 8*).

10. **“The government attempted to bring FGC to an end through both health and legislative approaches.” However, the fine is very less so people are not bothered about it. And I believe that because of less resources with the police, there might be hardly any enforcement. Any act or rule is just for the namesake if it is not supported by a strong regulatory and enforcement framework.**

**Response**:

We agree with this view and put it as a recommendation (*page 30, line 1 to 4*)

11. **“The Ethiopian Criminal Code has labeled FGC as an illegal act. Some news of
    prosecution and the threat of trial are also public.” The law should be enforced in a way that people understand it and are fearful. Simultaneously, awareness among girls, their mothers and other women, and other people who perform FGC, on the adverse effects of the FGC on girls and their next generations, should be done. Policymakers should be serious about the FGC, by putting more resources, amending the laws/ rules and be the role models.**

**Response**:

We agree with these views and recommended all of them in the conclusion and recommendation’ subsection’ (*page 30, 4 to 6*).

12. **Finally, the findings suggest that extensive public awareness creation, working with the local elders (men and women), and educating girls about its multi-faceted implications are more viable ways of ending the practice of FGC.” Role of religious leaders? People follow the churches where they should be told/or get the opportunity to discuss about the adverse effects of FGC on society.**

**Response**:

We agree with these views and recommended them in the conclusion and recommendation subsection (*page 30 line 4to 6*).

13. **IPPF, write full**

**Response**:

This and other abbreviations are written in full at the location where they first appear.

**C. Reviewer 2:**

1. **Reviewer 2 recommends the IMRAD style for our manuscript**

**Response**:

We have followed the IMRAD style in this manuscript.

2. **Some components of the ‘method section’ appear in the ‘introduction section’ of the manuscript.**

**Response**:

We have moved a brief description of the study site (Dawo District) from the ‘Introduction section’ to the ‘Method section’ under a new subsection ‘Study site’ (*page 20 line 15 to 21).*

3. **What was your study objective?**

**Response**:

A new sentence specifying the objective of the research is inserted (*page 10 line 11 to 13*)

4. **Which study design did you apply?**

**Response**:

We inserted a new subsection ‘Research design’ in ‘Methods and materials’ section (*page 10, line 11 to 13*)

5. **Which qualitative data analysis did you utilize?**

**Response**:

We have addressed this question in the section titled, ‘Methods and materials’ (page 13, line 1 to 3).

**Other changes:**

1. We have inserted subsections such as research site, research design, conclusion and recommendations based on the review comments on page *10, 11, and 29,* respectively.
2. In order to comply with the citation style of the journal, we have avoided all the names of the authors for the in-text citations and as a result, there are some changes in the structure of the sentences and paragraphs throughout the text without affecting the original meanings and claims.
3. We have made a thorough edition to maintain the consistency of the language. Some insertions were introduced for clarity, neatness, and selection of appropriate wording, including punctuation marks and phrases.
